# Micellar Electrokinetic Chromatographic Study of the Separation of an Aromatase Inhibitor and a Tryciclic Antidepressant in the Breast Cancer Treatment

**DOI:** 10.4137/aci.s939

**Published:** 2008-08-13

**Authors:** J. Rodríguez Flores, A.M. Contento Salcedo, L. Muñoz Fernández

**Affiliations:** Department of Analytical Chemistry and Foods Technology, University of Castilla-La Mancha, 13071 Ciudad Real, Spain

**Keywords:** micellar electrokinetic chromatography, letrozole, imipramine, breast cancer, antidepressant, ruggedness and human urine

## Abstract

Micellar electrokinetic chromatography (MEKC) was investigated for the simultaneous determination of letrozole, imipramine and their metabolites in human urine samples over a concentration range of therapeutic interest. Experimental parameters such as pH of the running electrolyte, sodium dodecylsulphate (SDS) concentration, borate concentration, voltage, etc were investigated. Under optimal conditions of 25 mM SDS, 15 mM borate buffer (pH 9.2), 15% 2-propanol, as background electrolyte; 28 kV and 40 °C, as voltage and cartridge temperature, respectively; resolution between the peaks was greater than 1.7. Before the determination, a solid phase extraction (SPE) procedure with a C_18_ cartridge was optimized. Good linearity, accuracy, precision, robustness and ruggedness were achieved and detection limits of 12.5 ng/mL for letrozole and its metabolite and 37.5 ng/mL, were obtained for imipramine and their metabolites. Real determinations of these analytes in two patient urines were carried out. Sensitivity achieved in this method is sufficient to perform kinetic studies in humans.

## Introduction

**Figure 1:**
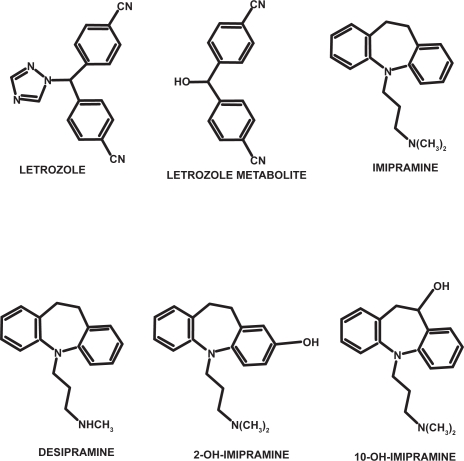
Chemical structures of letrozole, imipramine and their metabolites.

Many breast cancers rely on supplies of the hormone estrogen to grow. In women who have had their menopause, the main source of estrogen is through changing androgens (sex hormones produced by the adrenal glands) into estrogen; this occurs by an enzyme called aromatase. The conversion process is known as aromatization, and happens mainly in the fatty tissues of the body. Letrozole (LE) (trade name Femara^®^) is a drug that blocks the process of aromatization, and so reduces the amount of estrogen in the body; drugs that work in this way are known as aromatase inhibitors. The recommended therapeutic dose for LE is 2.5 mg per day. The major metabolic elimination route of LE is by to bis-4-cyanophenylmethanol (ME, its main metabolite) (Fig. 1). LE and its metabolite (ME) are excreted unchanged and their apparent elimination half-life time in plasma has been reported to be within approximately 2 days [1].

Often, mental disorders such as depression and anxiety may arise secondary to the presence of breast cancer and, it is therefore necessary to provide breast cancer patients with antidepressant treatment. Imipramine (IMI) is a tricyclic antidepressant (TCA) drug which is well absorbed in humans and undergoes extensive metabolism in the body. IMI is metabolized into a major active metabolite, desipramine (DES), which exhibits an activity profile similar to that of IMI and into two minor metabolites, 2-OH-imipramine (2-OH-IMI) and 10-OH-imipramine (10-OH-IMI). To establish pharmacokinetic parameters, it is therefore important to monitor urine concentration of IMI and all their metabolites.

About the analysis of LE and ME in biological fluids, to date, only a few methods have been reported. Marfil et al. published a high-performance liquid chromatographic (HPLC) method with a fully automated liquid-solid extraction and fluorescence detection that offers high sensitivity for the quantification of LE in plasma and urine, but not ME in either [2]. Mareck et al. reported a gas chromatography/mass spectrometric (GC-MS) method for determining these compounds; this method required a complex and long treatment of the urine with different steps, e.g. extraction, incubation of the sample for one hour, dryness evaporation, adding of organic solvent and finally a derivatization step [3]. Very recently, the authors of this work have also described a method to determine LE and ME in human urine by MEKC [4].

IMI and its demethylated metabolite (DES) have been determined in biological fluids using techniques such as HPLC coupled with ultraviolet and electrochemical detection [5–7], GC with nitrogen—phosphorus [8], mass spectrometric (MS) detection [9, 10] and capillary zone electrophoresis (CZE) [11]. Nonaqueous capillary electrophoresis (NACE) was used to separate tricyclic antidepressant drugs in biological samples after online solid phase extraction in pharmaceutical formulations [12] and plasma samples [13]. The simultaneous determination of IMI and all its metabolites was carried out by Chen et al. using HPLC [14].

Nevertheless, less effort has been put in the development of analytical methods that allow determination of LE along with antidepressants in biological fluids. These methods could be important because the joint administration of LE and antidepressants in patients with breast cancer, occurs frequently. Apparently, only one method has been published that uses MEKC for determining LE together with selective reuptake inhibitor (SSRIs) antidepressants and their metabolites in urine [15].

Capillary Electrophoresis (CE) can be regarded as an attractive separative technique due to its resolution, efficiency, ready optimization, widely variable operating conditions, short analysis time, use of small volumes of analytes and little waste solution.

The goals of this report were to develop a simple, fast and sensitive CE method that enables simultaneous determination of LE and one TCA (IMI) together with all its metabolites in human urine. This analytical method can be not only of interest in clinical toxicology, but also in forensics as often the metabolites of several drugs are involved in intoxications. Besides, this method can be employed to made pharmacokinetic studies in humans to understand the metabolic capacity and the synergy between both drugs.

## Experimental

### Apparatus

Analysis was performed with a Beckman P/ACE 5510 capillary electrophoresis system (Palo Alto, CA, USA), equipped with a diode array detector (DAD) and controlled by a Dell Dimension P133V computer with P/ACE station software. The 57 cm (50 to the detector) × 75 μm ID fused-silica separation capillary was maintained in a cartridge with a 100 μm × 800 μm detection window. The use of a photodiode detector allowed us to confirm the identity of the peaks, not only by its migration time, but also by the overlay of the UV-Vis spectra of the samples with a standard.

The extraction and pre-concentration processes were achieved with a Supelco (Bellefonte, PA, U.S.A) vacuum manifold coupled to a vacuum pump. The C_18_ cartridges were obtained from Waters (Milford, MA, U.S.A).

A Crison micro-pH 2002 instrument (Alella, Barcelona) was used for pH measurements.

Centrifugation of urine was carried out by a Selecta apparatus (Abrera, Barcelona).

### Chemicals and solutions

LE, IMI and their metabolites were kindly provided by Novartis Pharma (Basel, Switzerland).

Standard stock solutions (100 mg/L) of LE and ME were prepared in 50% (v/v) ethanol-water. Standard stock solutions (100 mg/L) of IMI, DES, 2-OH-IMI were prepared in water (Milli-Q quality). A standard solution (100 mg/L) of 10-OH-IMI was prepared in 50% (v/v) methanol-water. The resulting solutions were stored at 4 °C. Working standard solutions were prepared daily by diluting suitable aliquots of standard stock solution with Milli-Q water.

A 15 mM borate buffer (pH9.2) containing 25 mM SDS and 15% of 2-propanol was used as the background electrolyte and prepared daily.

All these reagents were from Panreac (Barcelona, Spain). All chemicals and solvents used were of analytical reagent grade.

### Operating conditions

Before first use the capillary was conditioned by flushing with 0.1 M NaOH for 20 min, then with water for 10 min, and finally with the background electrolyte solution for 10 min. The rinse step was performed by use of different vials from the separation vials, in order to keep the level of buffer in the anodic separation vial constant. At the start of each sequence the capillary was washed with 0.1 M NaOH for 3 min then with electrolyte separation buffer for 5 min, to restore the capillary wall surface and re-equilibrate the capillary between sample injections.

The sample vials were refrigerated at 8 °C inside the equipment. Separations were performed at 28 kV for 10 min at 40 °C. Under these conditions the current was 51 μA. Duplicate injections of the solutions were performed and average peak areas were used for the quantification.

### Treatment of the urine samples and SPE procedure

Fresh human urine samples were obtained from different volunteers who had or had not taken LE or IMI, and submitted directly to solid phase extraction after a preliminary centrifugation step (1398 g, 10 min, 20 °C).

The extraction of our compounds from the urine samples was performed in a reverse-phase C_18_ cartridge (Waters Sep-Pak Plus). The cartridge was preconditioned with 5 mL of methanol followed by 5 mL of 10 mM phosphate buffer solution (pH 7.0).

Different volumes (between 2 and 10 mL) of urine were slowly loaded into the conditioned cartridge. It was then washed with 8 mL of 10 mM phosphate buffer (pH 7.0) and 2 mL of a 20/80% (v/v) methanol-water solution. LE, IMI and their metabolites were eluted with 3 mL of methanol. Later on, this extract was evaporated to dryness with a gentle nitrogen stream and finally, they were reconstituted with 1 mL of Milli-Q water and transferred to the appropriate vials to be injected into the capillary electrophoresis equipment.

## Results and Discussion

### Optimisation of the MEKC procedure

Firstly, to achieve a suitable separation of the studied drugs, the effect of background electrolyte (BGE) pH (in the range 5 to 10) on resolution between peaks and analysis time was investigated using CZE. As BGEs were tested various salts (borate, phosphate, acetate,) to get buffer with different pH values. Baseline separation of IMI, LE and their metabolites was not achieved at any of the pH values arrayed. This was particularly predominant in the analysis of LE and their metabolites at all pH values; this is because they exist in a non-ionic form, and so MEKC was conducted for the separation of these drugs. SDS was used as surfactant in the separation electrolyte. In these preliminary studies it was observed that the best separation of our compounds was achieved at pH value of 9.2, which is provided by borate buffer solution. Therefore, this pH value was selected for the following studies.

#### Effect of SDS concentration

The effect of SDS concentration on migration times and resolution between peaks was researched over the range 10–40 mM maintaining constant pH (9.2), 15 mM borate buffer, as separation electrolyte, and 30 kV and 40 °C as separation voltage and cartridge temperature, respectively. 25 mM was selected as optimal SDS concentration. Lower concentrations of this surfactant provided shorter total analysis times but there were several peak overlaps, and higher SDS concentrations resulted in increased resolution between peaks but resulted in excessive analysis time was obtained.

#### Effect of ionic strength of electrolyte

The effect of the concentration of buffer solution (pH 9.2) from 5–40 mM on resolution of peaks and migration times was studied, with constant SDS concentration of 25 mM. A buffer concentration of 15 mM was selected as optimal because it renders good peak shapes, low current (51 μA) and better resolution between all peaks.

#### Effect of organic modifiers

**Figure 2:**
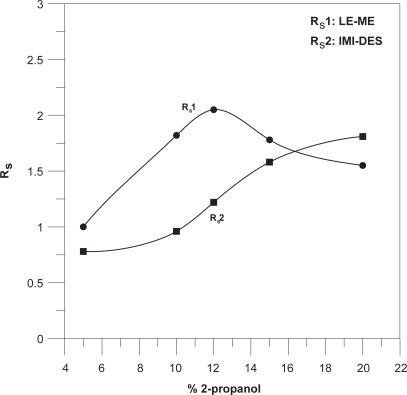
Effects of % 2-propanol on resolution between the peaks LE-ME and IMI-DES.

The addition of an organic modifier to the BGE may improve the selectivity and resolution; therefore the effects of several organic solvent (methanol, acetonitrile and 2-propanol) at different concentrations in the running buffer were examined. Elevated concentrations of solvent (>20%) were not used because these may cause the breakdown of micelles. When we used MeOH and ACN large and asymmetric peaks were obtained and did not allow the separation of all the studied compounds. Best selectivity, the most symmetric peaks, and the best resolution between peaks, was obtained at low 2-propanol concentration. Figure 2 shown the resolution between the peaks LE-ME, and IMI-DES over several concentration of 2-propanol, resolutions more affected by this parameters. 15% (v/v) 2-propanol was chosen optimal for resolution, analysis times and peak shapes.

#### Effect of voltage

The effect of the voltage applied from 10 to 30 kV was investigated. As expected, increasing the applied voltage, increases EOF, leading to shorter analysis time and higher efficiencies. A voltage of 28 kV yielded the best compromise for run time, separation current and resolution and was used for all experimental stages for the development of this method.

#### Effect of capillary temperature

**Figure 3:**
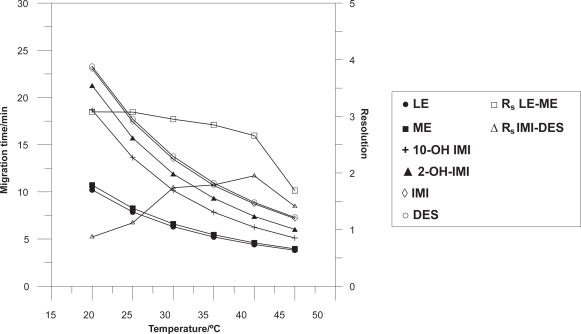
Influence of temperature on the migration times and peak resolutions. Operating conditions: 25 mM borate buffer pH 9.2, 25 mM SDS and 15% 2-propanol, separation voltage 28 kV, injection time 6 s, and detection at 205 nm.

Changes in capillary temperature can cause variations in efficiency, migration times and injection volumes. The effect of the temperature on the separation was investigated in the range of 20 to 45 °C. When, the temperature increases, the viscosity of buffer decreases, so the resistance of the buffer decreases and as the electric field is constant, the current increase. At higher temperatures, the migration time of all the studied compounds decreases (Fig. 3). 40 °C was selected because it provided the best resolution in the shortest total analysis time, and the generated current is lower than 52.8 μA.

#### Effect of injection time

To decrease the detection and quantification limits in the biological fluid studied (human urine), the injection times were investigated. This parameter was varied between 3 and 8 s at a constant pressure of 0.5 psi (1 psi = 6894.8 Pa).

As expected, when the injection time increased, the peak areas of all compounds also increased, but when this parameter was higher than 6s a loss of resolution was observed. For this reason, 6s was chosen for injection time.

### Solid Phase Extraction of the human urine samples (SPE)

Firstly, the electrophoretic procedure was applied to the analysis of human urine which had not been submitted to any special treatment; but due to the presence of large quantities of interfering compounds, and the low concentration of the studied compounds, it was necessary to carry out an extraction procedure for these compounds. Silica based apolar phases (C_18_) were used to extract LE, IMI and their respective metabolites; because this sorbent is of interest for the extraction of apolar compounds from a polar matrix such as urine. This cartridge has a silica-based bonded phase with strong hydrophobicity and was used to adsorb analytes of even weak hydrophobicity from aqueous solutions

Variables such as organic solvent; washing stages, using different solvents; organic solvent-water ratio, to elute our analytes free from interferences; and final volume of the extract were studied.

An improved electropherogram was obtained when loading the urine sample on the silica based apolar phases column previously washed with 8 mL of 10 mM phosphate buffer (pH 7.0) and 2.5 mL of a 20% methanol-water solution to minimize interferences. Finally, letrozole, imipramine and their metabolites were eluted with 3 mL methanol. Later on, this extract was evaporated to dryness with 1 mL of Milli-Q water and transferred to the appropriate vials to be injected into the capillary electrophoresis equipment. The maximal capacity of the silica based apolar phase column was investigated, and was determined to be 8 mL, therefore it was possible to preconcentrate eight times.

### Validation of the proposed electrophoretic procedure

#### Selectivity

**Figure 4:**
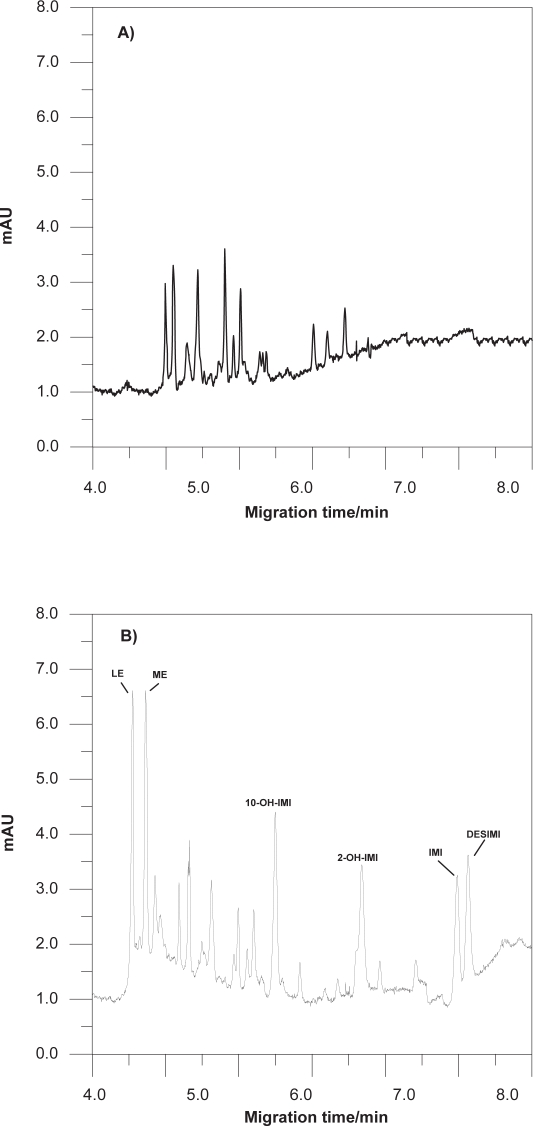
**A)** Blank of urine. **B)** MEKC electropherogram of a urine sample spiked with 3 mg/L of LE, IMI and metabolites. Operating conditions: hydrodynamic injection (6 s, 0.5 psi); separation: 15 mM (pH 9.2) borate buffer, 25 mM SDS and 15% 2-propanol, 28 kV as voltage of separation, 40 °C as capillary temperature and detection at 205 nm.

Representative electropherograms corresponding to the extracts from blank urine and urine spiked with 3 mg/L of each of the studied compounds are presented in Figure 4. At the migration times of our analytes, no interfering peaks of endogenous compounds were observed in blank human urine of the three volunteers.

Selectivity was also determined, by measurement of peak homogeneity using the techniques of normalization and comparison of spectra from different peak sections and absorbance at two wavelengths [16]. Both techniques proved to have a high level of purity of the peaks corresponding to the compounds studied in urine. Therefore, no interferences by matrix effect were observed.

#### Stability of the solutions

The stability of stock and diluted solutions of LE, IMI and their metabolites were investigated and results revealed that both solutions were stable for at least one month and seven days, for LE and IMI respectively.

The stability of LE, IMI and their metabolites at ambient temperature in urine samples were performed at several levels. The results revealed that all the compounds were stable for at least one day. It was confirmed that the repeated freeze and thawing of urine samples spiked with LE, IMI and their metabolites, did not affect the stability of these compounds.

#### Precision

To test the electrophoretic procedure suitability, eight injections of urine were spiked with LE, IMI and their metabolites, so that the concentrations at the end of the analytical pre-treatment were made 2.5 mg/L. The precision of the migration times and peak areas were very satisfactory with RSD between 0.26% and 0.81%, and 1.08% and 2.32% respectively, for all the studied compounds.

The operation for intermediate precision was repeated on different days and RSD values of less than 0.88% were obtained for migration times and less than 5.78% for peak areas. Comparison of the two sets of data with the aim of detecting random errors was carried out by applying the Snedecor F-test on these RSD values. Significant differences were not found in any case at a confidence level of 95%.

The precision of the total analytical procedure (including SPE) was evaluated by submitting six different spiked urine samples (2.5 mg/L for every compound) to this overall extraction-electrophoretic process (in duplicate). The results showed that RSD of the peak area for each compound was always less than 4.11%. Likewise, RSD obtained for the migration times were under 0.53%.

The results obtained for the repeatability, intermediate precision and precision of the total analytical procedure are summarized in Table 1 and expressed in terms of RSD.

#### Linearity

The linear behaviour was determined from duplicate injections of spiked human urine samples (previously submitted to the SPE treatment) at six different concentration ranging from 1.0 to 5.0 mg/L for every compound. The obtained linear regression equations and regression coefficients are presented in Table 2.

In order to confirm linearity according to the Analytical Methods Committee (AMC), [17] the test for “the lack of fit” was satisfactorily overcome.

#### Limits of detection and quantitation

The limits of detection (LODs) and quantification (LOQs) were calculated by measuring the noise in different blanks, and taking into account a factor of 3 and 10 for LODs and LOQs, respectively, and by using standards obtained in order to convert to concentration units. The LODs and LOQs have been calculated taking into account the overall process (extraction, preconcentration and MEKC step), and by passing through the SPE column 8 mL of urine samples (Table 2).

#### Accuracy

The accuracy expresses the closeness of agreement between the value found and the value that it is accepted as a reference value. In order to test the accuracy of the proposed method, several aliquots of standard solutions of LE, IMI and their metabolites were added into human urine samples. These samples were analysed using the extraction, pre-concentration and electrophoretic procedures described in this work. The concentrations found in the test solutions were then calculated by reference to the duplicate bracketing standard solutions and the recoveries obtained are summarized in the Table 3.

#### Integral robustness-ruggedness evaluation

The United States Pharmacopeia (UPS) defines ruggedness as “the degree of reproducibility of the test results obtained by the analysis of the same samples under a variety of normal test conditions such as different days, several reagent lots, different lots, different instruments, various laboratories, different elapsed assay times…” where all of these factors are external to the written analytical method.

The robustness of a method is defined by both the USP and ICH Tripartite guidelines as “a measure of its capacity to remain unaffected by small but deliberate variations in method parameters and provides an indication of its reliability during normal use [18]”. Ruggedness can therefore be regarded as a measure of the absence of external influences on the test results, whereas robustness measures the lack of internal influences on these results.

In this work we have tested the influence of variations in both internal and external parameters of the method (e.g. pH and ionic strength of buffer, SDS concentration, voltage, capillary temperature, different days for analysis, etc), whose influence has been studied at different levels.

The Plackett-Burman fractional factorial model, which is based on balanced incomplete blocks, was employed to evaluate this aspect of the method.

For statistical reasons (concerning effects on interpretation), designs with fewer than eight experiments are not used, while those with more than 24 experiments were considered unpractical [19, 20]. To date, this model has been usually satisfactorily applied just in the evaluation of robustness.

A novel Plackett-Burman design that involves the evaluation of both robustness and ruggedness effects (eleven factors and twelve experiments, N = 12) is presented in Table 4 (http://www.locumusa.com/pdf/general/article01.pdf). The choice of variables (factors) and the levels at which they are tested is very important for a reliable robustness/ruggedness test. In our case, the variables selected as factors are instrumental and chemical parameters that are significant in the performance of the proposed method. The selection about the levels of these factors should reflect slight variations, which could be usually observed. The external (ruggedness) and internal (robustness) factors (A-K) selected for our model are presented in Table 5, which also shows the (+) and (−) levels for every factor that are, respectively, upper and lower values with regard to optimal in the procedure.

The effects of varying the levels on the most critical electrophoretic responses of the method were investigated.

The “ranked effects” (main effects calculated according to Plackett and Burman) for every selected factor on a specific electrophoretic response were calculated by simple addition of its (−) and (+) assay test results, upon the design shown in Table 4. The total result obtained for every factor was divided by half the number of samples. The M values are statistic constants for any given design table with a number of elements; there are eleven factors in our case [21]. Finally, the obtained ranked effects for the 11 selected factors were plotted (on the x-axis, in increasing order) against the M values (on the y-axis) for each critical electrophoretic response. The results from this plot must be near to a straight line. If a value lies outside this straight line, it can be concluded that the method is not rugged/or robust at this point (as classified by its corresponding factor). However, if the results from the plot form a (nearly) straight line, it can be concluded that the analytical method is rugged and robust over the conditions tested in the run design.

The robustness/ruggedness evaluation was performed in our case, by carrying out duplicate injections of urine samples spiked with 3 mg/L of all compounds. The described robustness/ruggedness test showed our SPE-electrophoretic method to be both robust and rugged for the critical electrophoretic responses assessed for all the variations tested in this study.

**Figure 5:**
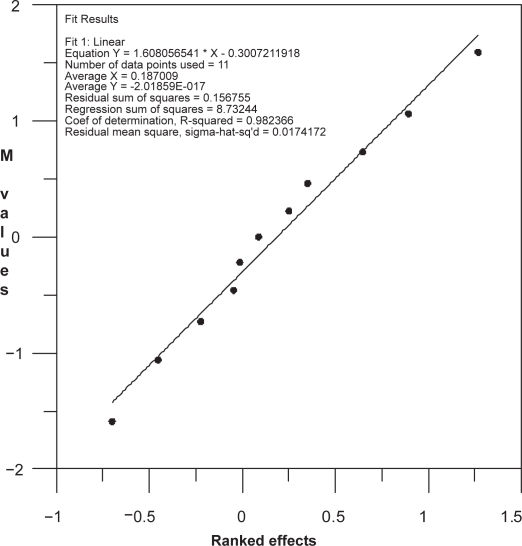
Plot corresponding to M values for the resolution R_s_ between IMI and DES vs. ranked effects of the 11 selected factors.

As an example, the plot corresponding to the ranked effects of the 11 selected factors vs. M values for the resolution between IMI and DES, is shown in Figure 5. It can be seen from this plot that all the points lie on a straight line and, therefore, our analytical method can be considered robust and rugged about this electrophoretic response.

In general terms, the described robustness/ruggedness test showed our electrophoretic method is both robust and rugged enough for the critical electrophoretic responses; being assessed for all the variations tested in this study.

## Analysis of Human Urine Samples

To demonstrate the applicability of the extraction, preconcentration and MEKC procedure developed in this report, urine samples of two volunteers (patients undergoing medical treatment) were analysed.

Then, urine of two different volunteers undergoing medical treatment with LE or IMI were analysed. The first is a woman A, that receives 2.5 mg per day of LE orally (year 3 of treatment), and the second is a woman B, takes 25 mg per day of IMI orally (recent commencement of treatment). The urine samples were collected up to 7 hours after oral single dose administration of LE (woman A) or 5 hours after oral single dose administration of IMI (woman B). The concentrations of each compound found in the analyzed urine samples are shown in Table 6. All determinations were carried out in duplicate. To evaluate the possible matrix effect, the method of standard addition was used for the determination of these compounds in some urine samples, the results obtained are also shown in Table 6. As shown, these results coincide with those obtained by direct measurement by the proposed method.

**Figure 6:**
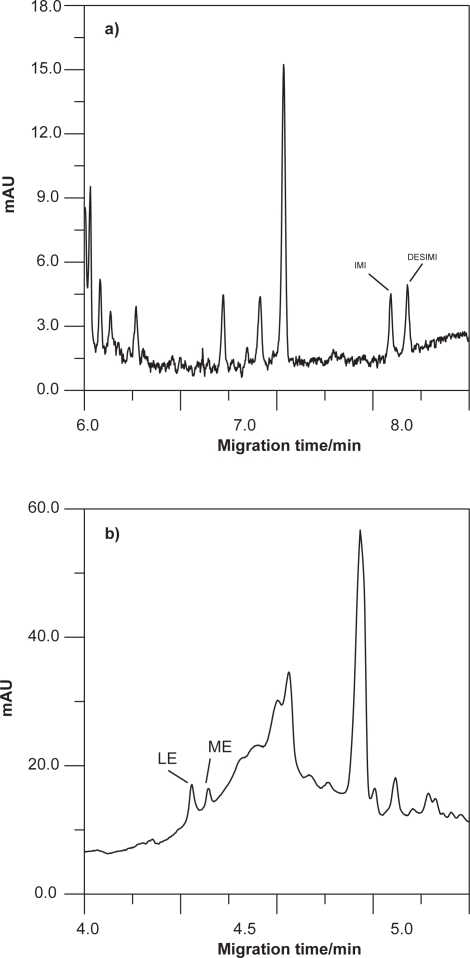
Electropherogram corresponding: **a)** urine sample from a woman on imipramine treatment (recently treatment) after 5 h of the administration of 50 mg dosage, **b)** urine sample from a woman on letrozole treatment (3 year of treatment) after 7 h of the administration of 2.5 mg dosage.

Two electropherograms of these experiments under conditions optimised in this paper are shown in Figure 6.

## Conclusions

A simple, specific and sensitive MEKC method has been developed for the analysis of LE, IMI, and their metabolites in human urine. Although, LE and IMI have previously been determined independently, this is the first report that enables the determination of LE and its main metabolite, along with IMI and their metabolites, together, in human urine. MEKC proved to be an effective technique for the simultaneous analysis of these drugs.

Before MEKC analysis, a reproducible SPE method for LE, IMI and their metabolites was developed, which permits the quantification of these compounds at clinical concentration. The method provided excellent linearity, accuracy, specificity, sensitivity, precision and ruggedness.

## Figures and Tables

**Table 1:** Precision.

**Compounds**	**Repeatability^a^RSD(%), n = 8**		**Intermediate precision^a^RSD(%), n = 16**		**Precision^b^SD(%), n = 6**	

	t_m_^*^	PA^**^	t_m_^*^	PA^**^	t_m_^*^	PA^**^
LE	0.26	1.74	0.44	5.07	0.36	1.60
ME	0.81	1.55	0.62	2.53	0.24	2.02
10-OH-IMI	0.40	1.79	0.86	5.35	0.42	3.43
2-OH-IMI	0.38	2.31	0.88	4.67	0.53	3.16
IMI	0.42	1.08	0.74	4.20	0.51	1.94
DES	0.38	2.32	0.71	5.78	0.50	4.11

(*) t_m_, migration time; (**) PA, peak area.

a) Relative to the single electrophoretic procedure.

b) Precision of the total analytical procedure (including the SPE).

**Table 2:** Linearity, LODs and LOQs values.

**Peaks**	**Equation**	**Regresion coefficient**	LODs^*^ (ng/mL)	LOQs^**^ (ng/mL)
LE	Y = (2908.0 ± 195.0) × −(718.7 ± 601.9)	0.982	12.5	37.5
ME	Y = (2811.9 ± 252.8) × −(247.3 ± 117.9)	0.989	12.5	37.5
IMI	Y = (2056.3 ± 110.8) × −(148.3 ± 141.1)	0.991	37.5	112.5
DES	Y = (1851.6 ± 32.9) × −(110.7 ± 101.3)	0.998	37.5	112.5
2-OH-IMI	Y = (1553.0 ± 50.5) × −(183.9 ± 112.5)	0.985	37.5	112.5
10-OH-IMI	Y = (2008.8 ± 40.12) × −(156.7 ± 101.0)	0.997	37.5	112.5

* LODs: limits of detection.

** LOQs: limits of quantification.

**Table 3:** Accuracy of human urine samples.

**Compounds**	**Sample 1**		**Sample 2**		**Sample 3**		**Sample 4**	

	**Added (mg/L)**	Recoveries (%)*	**Added (mg/L)**	Recoveries (%)*	**Added (mg/L)**	Recoveries (%)*	**Added (mg/L)**	Recoveries (%)*
LE	1.5	96.31 ± 0.05	2.5	102.90 ± 0.08	3.5	100.43 ± 0.03	4.5	98.50 ± 0.02
ME	1.5	102.20 ± 0.02	2.5	95.52 ± 0.01	3.5	99.41 ± 0.09	4.5	100.82 ± 0.07
IMI	1.5	99.50 ± 0.01	2.5	102.53 ± 0.03	3.5	102.12 ± 0.11	4.5	101.50 ± 0.02
DES	1.5	104.84 ± 0.06	2.5	97.80 ± 0.05	3.5	98.10 ± 0.03	4.5	99.73 ± 0.05
2-OH-IMI	1.5	94.91 ± 0.03	2.5	94.21 ± 0.06	3.5	95.37 ± 0.01	4.5	98.21 ± 0.03
10-0H-IMI	1.5	96.23 ± 0.04	2.5	97.60 ± 0.10	3.5	100.82 ± 0.05	4.5	102.30 ± 0.08

^*^ n = 3.

**Table 4:** Experimental design for the whole robustness-ruggedness evaluation using the Plackett-Burman model.

**Experiment number**			**External/internal changes or variations (11)**								
	**Selected factors**										
	A	B	C	D	E	F	G	H	I	J	K
1	+	+	−	+	+	+	−	−	−	+	−
2	−	+	+	−	+	+	+	−	−	−	+
3	+	−	+	+	−	+	+	+	−	−	−
4	−	+	−	+	+	−	+	+	+	−	−
5	−	−	+	−	+	+	−	+	+	+	−
6	−	−	−	+	−	+	+	−	+	+	+
7	+	−	−	−	+	−	+	+	−	+	+
8	+	+	−	−	−	+	−	+	+	−	+
9	+	+	+	−	−	−	+	−	+	+	−
10	−	+	+	+	−	−	−	+	−	+	+
11	+	−	+	+	+	−	−	−	+	−	+
12	−	−	−	−	−	−	−	−	−	−	−

−, +: Levels for the factors.

**Table 5:** Variables selected as factors and values chosen as levels.

**Factors**	**External/internal**	**Optimal**	**Level (−)**	**Level (+)**
(A) Different days	External	–	2	1
(B) Different buffers	External	–	2	1
(C) Different patiens	External	–	2	1
(D) MeOH(%) washing	Internal	20	18	22
(E) Elution volume (SPE)	Internal	3	2.8	3.2
(F) Injection time (s)	Internal	6	5	7
(G) Voltage (kV)	Internal	28	26	30
(H) [Buffer] (mM)	Internal	15	13	17
(I) [SDS] (mM)	Internal	25	23	27
(J) Percent 2-propanol	Internal	15	13	17
(K) λ detection (nm)	Internal	205	203	207

**Table 6:** Analysis of human urine samples.

	**Woman A**		**Woman B**	

	**Standard addition (mg/L)**	**Direct measurement (mg/L)**	**Standard addition (mg/L)**	**Direct measurement (mg/L)**
LE	0.40	0.40	–	–
ME	0.16	0.15	–	–
IMI	–	–	0.55	0.56
DES	–	–	0.61	0.61
10-OH-IMI	–	–	*	*
2-OH-IMI	–	–	*	*

^*^ Amount detected but not quantified.

## Abbreviations

MEKC: Micellar Electrokinetic Chromatography
LE: letrozole, ME, bis-4-cyanophenylmethanol (letrozole’s main metabolite)
IMI: imipramine
DES: desipramine
10-0H-IMI: 10-OH-imipramine
2-OH-IMI: 2-OH-imipramine
SDS: sodium dodecylsulphate
SPE: (solid phase extraction)
